# *FASEB BioAdvances* announces changes in 2024

**DOI:** 10.1096/fba.2024-00043

**Published:** 2024-03-20

**Authors:** Loren E. Wold, Crislyn D'Souza‐Schorey, Yung Hou Wong

**Affiliations:** ^1^ College of Medicine, The Ohio State University Columbus Ohio USA; ^2^ University of Notre Dame Notre Dame Indiana USA; ^3^ The Hong Kong University of Science and Technology Hong Kong China

In 2019, The Federation of American Societies for Experimental Biology (FASEB) started publishing *FASEB BioAdvances* as a fully open‐access partner to its flagship *FASEB Journal* for authors to publish their quality research spanning the breadth of the biological and biomedical sciences. Today, the journal publishes a variety of manuscript types, including original research, reviews, and perspectives on current issues in science and academia. The scope of *FASEB BioAdvances* overlaps with *The FASEB Journal*, and although its priority is also quality science, it puts less emphasis on perceived impact (e.g., is a sound science journal) and now also welcomes manuscript types that we believe will improve rigor and reproducibility, including replication studies or reports on negative (null) findings.

Drs. Crislyn D'Souza‐Schorey and Yung Hou Wong have served as Editors‐in‐Chief since January 2022. During their tenure, they have focused their efforts on timely review and publication of cutting‐edge science, cross‐disciplinary science, and original research. Their efforts have repositioned the journal with a focus that serves it well into the future.

Recently, FASEB considered how to best grow published output in *FASEB BioAdvances* without reducing quality or compromising on scientific integrity. FASEB's analysis also identified a need for a seamless process for authors transferring manuscripts from *The FASEB Journal* to *FASEB BioAdvances* in a way that further strengthens their partnership. In an effort to increase the value of *FASEB BioAdvances* to its authors, to decrease the time to first decision, and to better align with the needs of the global author community served by FASEB, we are announcing a change in the editorial structure of the journal. Since February 2024, *FASEB BioAdvances* is under the direction of Loren E. Wold, PhD, Editor‐in‐Chief of *The FASEB Journal* who will work to strengthen both journals. Dr. Wold and team are working on enhancing manuscript transfer options for authors, adding a new dedicated Referral Editor to work between both journals to be a resource for authors, and a plan to introduce greater emphasis on the publication of thematic special collections in areas of considerable importance. *The FASEB Journal* is fortunate to already have in place a team of over 200 dedicated and diverse researchers: a Senior Editor, a team of eight Associate Editors, and a Special Issues and Reviews Editor, an almost 100‐member editorial board, and a 116‐member early career researcher editorial board. The *FASEB BioAdvances* Deputy Editor will continue to serve in that role and will join *The FASEB Journal* editorial team. Associate Editors of *FASEB BioAdvances* will continue to serve in their roles. Leveraging this new, broad, and expanded editorial team for both journals will be an important advantage for authors by ensuring rapid review, editing and publishing of sound high‐quality science, and seamless transfers of manuscripts between journals.

*Postscript*: As the incoming Editor‐in‐Chief and on behalf of FASEB, Loren shares a grateful thank you with Drs. D'Souza‐Schorey and Wong for their dedication and outstanding work with *FASEB BioAdvances*, which has greatly improved the author experience, led to the publication of outstanding science, and repositioned the journal as research‐focused and on a bright path. Authors worldwide who are seeking a journal home will find one with FASEB. We are committed to high‐quality rapid peer review, scientific integrity with tools to support authors, a diverse editorial team, broad citations, and a global readership for your research. We welcome your submissions.

## Data Availability

Stored in repository.

